# Time to Exhale: Additional Value of Expiratory Chest CT in Chronic Obstructive Pulmonary Disease

**DOI:** 10.1155/2018/9493504

**Published:** 2018-03-04

**Authors:** Joshua Gawlitza, Frederik Trinkmann, Hans Scheffel, Andreas Fischer, John W. Nance, Claudia Henzler, Nils Vogler, Joachim Saur, Ibrahim Akin, Martin Borggrefe, Stefan O. Schoenberg, Thomas Henzler

**Affiliations:** ^1^Institute of Clinical Radiology and Nuclear Medicine, University Medical Center Mannheim, Medical Faculty Mannheim, Heidelberg University, Heidelberg, Germany; ^2^Department of Medicine (Cardiology, Angiology, Pulmonary, and Intensive Care), University Medical Center Mannheim, Medical Faculty Mannheim, Heidelberg University, Heidelberg, Germany; ^3^Department of Radiology and Radiological Science, Medical University of South Carolina, Charleston, SC, USA; ^4^DZHK (German Center for Cardiovascular Research), Partner Site Mannheim, Berlin, Germany

## Abstract

**Objectives:**

Diagnostic guidelines for chronic obstructive pulmonary disease (COPD) are based on spirometry and clinical criteria. However, this does not address the pathophysiological complexity of the disease sufficiently. Until now, inspiratory chest computed tomography (CT) has been considered as the preferred imaging method in these patients. We hypothesized that expiratory CT may be superior to demonstrate pathophysiological changes. The aim of this prospective study was to systematically compare lung function tests with quantified CT parameters in inspiration and expiration.

**Materials and Methods:**

Forty-six patients with diagnosed COPD underwent spirometry, body plethysmography, and dose-optimized CT in maximal inspiration and expiration. Four quantified CT parameters were acquired in inspiration, expiration, and their calculated delta values. These parameters were correlated with seven established lung function parameters.

**Results:**

For inspiratory scans, a weak-to-moderate correlation with the lung function parameters was found. These correlations significantly improved when adding the expiratory scan (*p* < 0.05). Moreover, some parameters showed a significant correlation only in expiratory datasets. Calculated delta values showed even stronger correlation with lung function testing.

**Conclusions:**

Expiratory quantified CT and calculated delta values significantly improve the correlation with lung function parameters. Thus, an additional expiratory CT may improve image-based phenotyping of patients with COPD.

## 1. Introduction

Chronic obstructive pulmonary disease (COPD) is a common and largely avoidable disease that is characterized by irreversible airway obstruction, predominantly due to inhaled noxae and particles. COPD was listed as the third leading cause of death by the World Health Organization in 2012 and has surpassed epidemiological estimations by the Global Burden of Disease Project, now causing over 3.1 million deaths per year [1, 2].

Traditionally, the diagnosis of COPD is based on spirometric measurements of forced expiratory volume in one second (FEV_1_) and forced vital capacity (FVC), as specified by the Global Initiative for Chronic Obstructive Lung Disease (GOLD) [3]. However, recent studies have shown several shortcomings of this approach. While Hardie et al. showed a risk of overdiagnosis of COPD among the elderly using common spirometry criteria, Cerveri et al. were able to demonstrate an underestimation of airflow obstruction among young adults when using spirometric measurements [4, 5]. Other pulmonary function tests, such as multiple-breath washout, provide a better differentiation of healthy controls and COPD patients, even with preserved lung function in spirometry [6–8].

Chest computed tomography (CT) is currently not listed as an obligate diagnostic tool in patients with suspected COPD by GOLD [3]. Nevertheless, imaging provides additional information as compared to spirometry by detection of pathological changes that directly contribute to the airflow limitations [9]. Emphysema, bronchial wall thickening, and air trapping are the key pathologic findings of COPD on chest CT that are associated with increased mortality [9–12]. Moreover, fibrotic changes of the lungs, which have significant symptom overlap with COPD, can only be differentiated from COPD using imaging [13].

Despite these benefits, CT has not become a recommended examination in newly diagnosed COPD yet since the clinical impact is not fully understood [9]. In this context, the American Thoracic Society and the European Respiratory Society proposed to evaluate the role of routine chest CT [14].

Several prior studies have correlated quantified chest CT measurements with functional lung parameters [15–17]. However, most studies to date have focused on the correlation of spirometry and quantified CT. In addition to the previously described limitations, spirometry does not provide residual volume, which is pathologically altered in COPD. Another limitation of recent studies is the quantified CT acquisition itself, which is most frequently performed only during maximal inspiration [16]. COPD mainly limits airflow during expiration, possibly limiting the diagnostic yield of inspiratory-only protocols; accordingly, the Fleischner Society has suggested the potential additive value of expiratory acquisitions [18].

Thus, the aim of this prospective study was to investigate the correlation of functional lung parameters beyond FEV_1_ and FVC with quantified CT parameters acquired during maximum inspiration as well as maximum expiration.

## 2. Materials and Methods

### 2.1. Subjects

The HIPAA compliant study protocol, which is in accordance with the Declaration of Helsinki, was approved by our local ethics committee (*blinded for review*). The study was registered at http://www.clinicaltrials.gov (*blinded for review*).

We prospectively enrolled 49 patients with previously diagnosed COPD and a clinical indication for unenhanced chest CT in a single-center, all-comer approach. Written informed consent was obtained from all patients following a full explanation of the purpose of the study and of potential risks and discomforts associated with their participation.

### 2.2. Study Protocol

#### 2.2.1. Lung Function Testing

All patients underwent spirometry and whole-body plethysmography (MasterScreen® Body, CareFusion, Höchberg, Germany) yielding the following parameters: vital capacity (VC), FEV_1_, Tiffeneau index (FEV_1_%VC), residual volume (RV), total lung capacity (TLC), ratio of residual volume to TLC (RV%TLC), and specific total airway resistance (sR_tot_). Except for FEV_1_%VC and RV%TLC, all values are given as percent of predicted, as calculated according to current ATS/ERS recommendations or GLI equations, respectively [19, 20].

#### 2.2.2. CT Examinations

A noncontrast chest scan was performed in maximum inspiration and maximum expiration using a 3rd generation dual-source CT (Somatom FORCE, Siemens Healthineers, Forchheim, Germany) at 100 kVp with a dedicated tin filter for dose reduction [21]. The scan parameters were as follows: 100 kVp tube voltage, 96 mAs reference tube current using automated tube current modulation (effective mAs = 166.5 ± 105), 0.25 s rotation time, pitch 1.2, and 192 mm × 0.6 mm detector collimation. All images were reconstructed with a slice thickness of 1.5 mm, using a dedicated reconstruction kernel for quantitative lung analysis (Br32) and a novel iterative reconstruction technique (Adaptive Model-based Iterative Reconstruction (ADMIRE), Siemens Healthineers, Germany). The algorithm of ADMIRE was substantially explained in a recent work [22]. An iterative level of four was chosen for the present study as recommended by the CT vendor for quantitative lung analysis. The average CTDI (computed tomography dose index) was 0.48 ± 0.19 mGy and the mean DLP (dose length product) 17.2 ± 6.5 mGy·cm.

#### 2.2.3. Image Analysis

**Figure 1:**
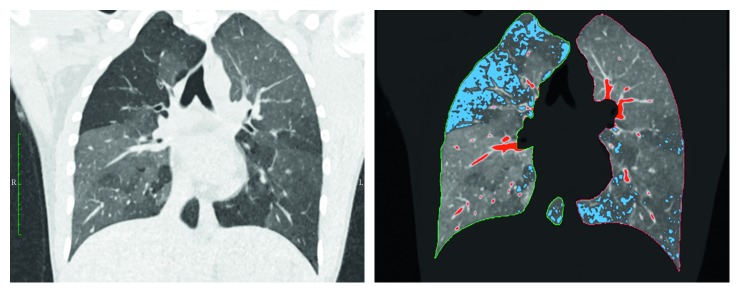
Automatic detection of lung borders and lung parenchyma. Blue areas: low attenuation volume (LAV) with HU values below −950; red areas: high attenuation volume (HAV) with HU values above −200.

Inspiratory and expiratory datasets were analyzed using dedicated semiautomatic software (SyngoViaVB10, Pulmo3D, Siemens Healthineers, Forchheim, Germany). Lung segmentation was automated and manually revised if necessary (Figure 1). Four quantitative parameters were acquired: total lung volume (volume), mean lung density (MLD), full width half max (FWHM), and low attenuation volume (LAV). The LAV threshold for emphysema was set to −950 HU. This cutoff had been extensively evaluated in previous studies and strongly correlates with microscopic and gross emphysema [23–25]. FWHM marks the width at the half maximum of the voxel count to specific HU value curve (voxel-density histogram) representing the density distribution of the lung parenchyma. An exemplary voxel-density histogram with its corresponding FWHM can be found in the Supplementary Materials (available here). The difference in the values between inspiratory and expiratory scans was defined as delta value.

### 2.3. Statistical Analysis

A total of 28 correlation pairs (four quantified CT parameters and seven lung function parameters) were analyzed for inspiratory, expiratory, and delta values. The Pearson product-moment correlation coefficient was calculated for each pair using JMP 11 (SAS, Cary, USA). The correlation coefficients of inspiratory and expiratory scans were compared by Pearson and Filon z test, using cocor Software [26]. Based on previously published data, we assumed a correlation of *r*=−0.252 between LAV and FEV_1_ [27]. Based on this correlation, we calculated that a planned sample size of 34 patients would give the study a power of 90% at a five percent significance level to detect a significant correlation. A *p* value of less than 0.05 was considered statistically significant.

## 3. Results

The study population consisted of 46 patients (26 male) with previously diagnosed COPD. Twenty patients were active smokers at the date of examination. The remaining 26 patients had smoked in the past (Tables 1–3).

**Figure 2:**
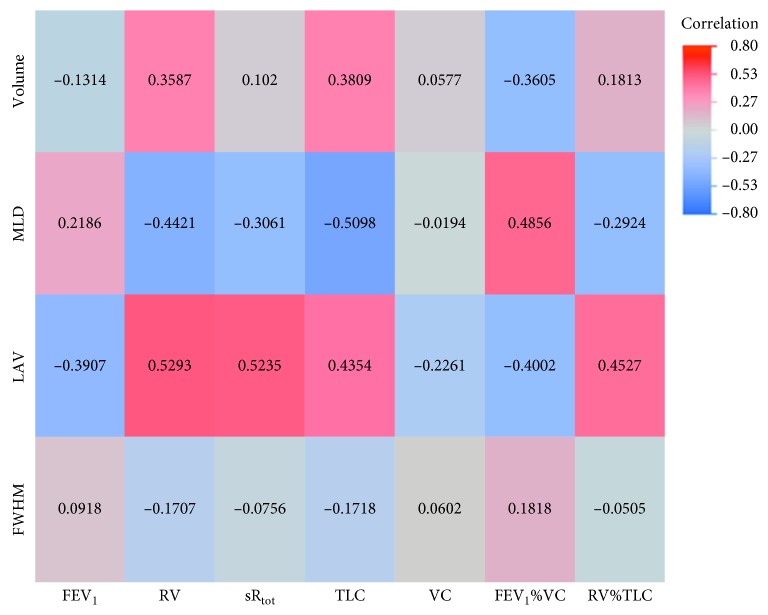
Heat map of correlations for inspiratory values. MLD: mean lung density; FWHM: full width half max; LAV: low attenuation volume; VC: vital capacity; FEV_1_: forced expiratory volume in one second; FEV_1_%VC: Tiffeneau index; RV: residual volume; TLC: total lung capacity; sR_tot_: specific total airway resistance.

**Figure 3:**
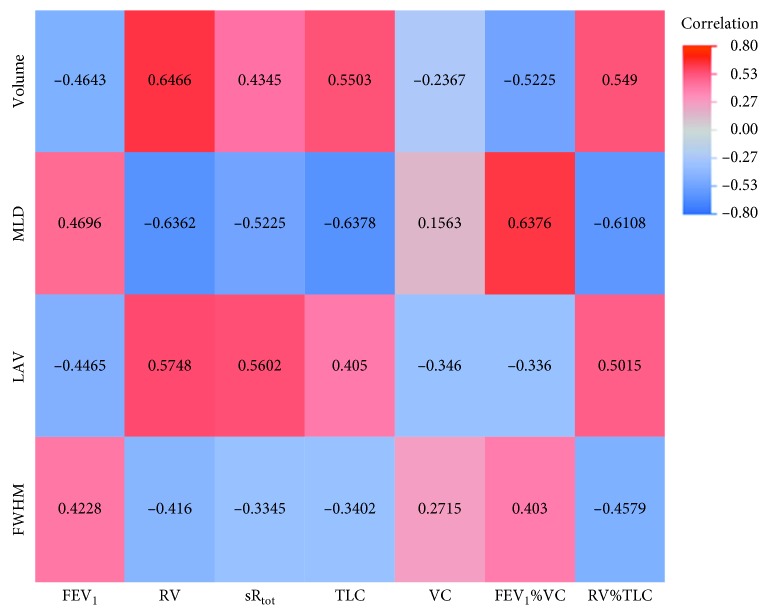
Heat map of correlations for expiratory values. MLD: mean lung density; FWHM: full width half max; LAV: low attenuation volume; VC: vital capacity; FEV_1_: forced expiratory volume in one second; FEV_1_%VC: Tiffeneau index; RV: residual volume; TLC: total lung capacity; sR_tot_: specific total airway resistance.

**Figure 4:**
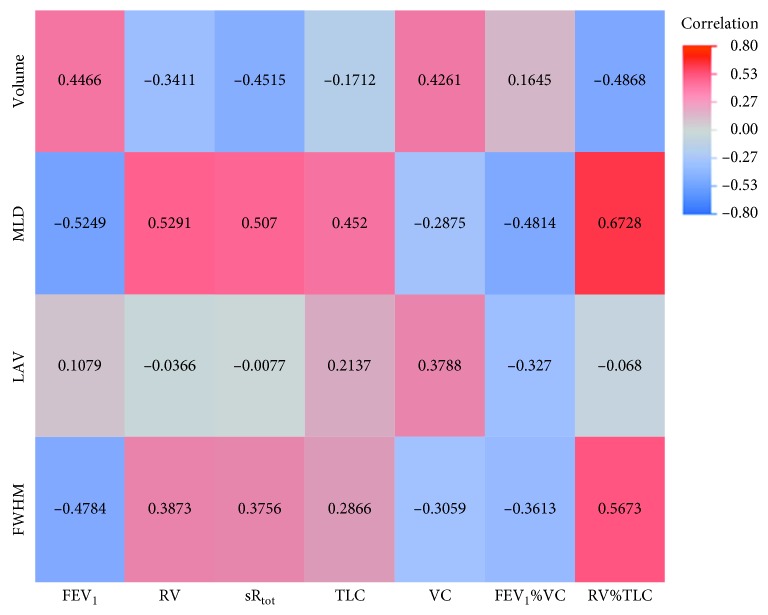
Heat map of correlations for delta values. MLD: mean lung density; FWHM: full width half max; LAV: low attenuation volume; VC: vital capacity; FEV_1_: forced expiratory volume in one second; FEV_1_%VC: Tiffeneau index; RV: residual volume; TLC: total lung capacity; sR_tot_: specific total airway resistance.

Regarding inspiratory, expiratory, and delta values, we were able to show statistically significant correlations between every quantified CT parameter and each lung function parameter in either of one of the analyses (inspiration, expiration, and delta values; Tables E1–E3 in the Supplementary Materials). However, there was a strong difference in correlation distribution between quantitative parameters from the inspiratory and the expiratory CT scan as well as the delta values, as seen in the correlation heat maps (Figures 2–4). As substantiated in Figure E1 in the Supplementary Materials focusing on MLD, expiratory and delta parameters show improved correlation with spirometric data as compared to inspiratory parameters.

### 3.1. Inspiratory Quantified CT Values

The fewest significant correlations were found when using data from the inspiratory acquisition (14 out of 28 correlated pairs; Table E1 in the Supplementary Materials). The significant correlations of inspiratory values to lung function values had a range from −0.5098 to 0.5293 (*p* values 0.0415 to 0.0001). The strongest correlation was found between MLD and RV (*r*=0.5293; CI: 0.2885 to 0.7071; *p*=0.0001). No single significant correlation between FWHM and the functional lung parameters could be shown. No quantified CT parameter from the inspiratory scan correlated with VC.

### 3.2. Expiratory Quantified CT Values

The most significant correlations were found when using data from the expiratory acquisition (25 out of 28 correlated pairs; Table E2 in the Supplementary Materials). As visualized in Figures 2 and 3, the underlying correlation pattern is equal to the inspiratory values. Correlation coefficient analysis showed 17 significantly higher correlations in the expiratory scan compared to the inspiratory scan (Table 4). Every quantified CT parameter correlated with every functional lung parameter except for VC. Only LAV showed a significant negative correlation to VC (*r*=−0.346; CI: −0.5717 to −0.0718; *p* value = 0.0149). Overall, the significant correlations had a range from −0.6378 to 0.6466 (*p* values: 0.0188 to <0.0001). The strongest correlation was found between total volume and RV (*r*=0.6466; CI: 0.444 to 0.7863; *p* value < 0.0001).

### 3.3. Delta-Quantified CT Values

As seen in Figure 4, the correlations for the delta values are contrary to the ones for the inspiratory and expiratory values. Twenty-one correlation pairs showed a significant correlation in the calculated quantified CT delta values (Table E3 in the Supplementary Materials). The correlations had a range from −0.5098 to 0.6728 (*p* values: 0.8049 to <0.0001). In contrast to the inspiratory and the expiratory values, every quantified CT parameter showed a correlation to VC. The strongest correlation was found between MLD and RV%TLC (*r*=0.6728; CI: 0.483 to 0.8022; *p* value < 0.0001). The LAV did not correlate with any lung parameters for the delta-quantified CT values.

## 4. Discussion

Our study found significant correlations for quantified CT parameters and functional lung parameters beyond the commonly used FEV_1_ and VC. The additional scan performed in end-expiration showed overall stronger correlations compared to the inspiratory scan. These findings were consistent for both static and dynamic lung function parameters.

Every quantified CT parameter significantly correlated with the functional lung parameters in all of the three different analyses. Nevertheless, there was a strong difference in the extent and the number of significant correlations between the inspiratory, expiratory, and delta of the quantified CT parameters.

Stronger correlations were found between static parameters of lung volume, such as TLC and RV, as compared to the dynamic parameters FEV_1_, FEV_1_%VC, and sR_tot_. This finding could be reasonably expected, as the acquisitions were also static and provided predominantly anatomic information. Likewise, LAV correlated with these static parameters on all three analyses (i.e., there was no additive value of the expiratory scan or delta values), which can be expected, given the LAV was relatively fixed between inspiratory and expiratory acquisitions.

Our findings align with the 2015 Fleischner Society statement on CT-definable subtypes of COPD, in which they noted the potential additive information of end-expiratory acquisitions in patients with COPD [18]. A combination of inspiratory and expiratory quantified CT values has already been shown to correlate well with air trapping and COPD grading [15, 28]. Nevertheless, neither inspiratory nor expiratory scans significantly correlate with VC. These findings are in accordance with the weak correlations of VC and LAV found by Timmins et al. [29]. Only the calculated delta values showed a correlation with VC, although they do have limitations. Delta LAV values did not show significant correlations with lung function parameters. This was expected due to the previously mentioned constancy of the LAV in inspiratory and expiratory scans (i.e., delta values were very small). Several studies have already correlated ratios of inspiratory and expiratory quantified CT scans with functional lung parameters. For example, Nambu et al. demonstrated a correlation between the MLD ratio, calculated from inspiratory and expiratory scans, and functional lung parameters such as FEV_1_ [30]. Schroeder et al. compared emphysema and bronchial wall thickness to spirometry and found a strong correlation between the percentage of emphysema and FEV_1_ as well as FEV_1_/FVC [31]. The SPIROMICS investigators showed a correlation of small airway abnormalities and emphysema with FVC, FEV_1_, and FEV_1_/FVC among 580 individuals between the ages of 40 and 80 [32]. Decreased MLD is associated with hyperinflation and structural damage caused by COPD and correlates with parameters of functional emphysema such as RV or RV%TLC. Correlations of the latter steadily increase from inspiratory to expiratory to delta values. The strongest relation of the dynamic parameters FEV_1_, FEV_1_%VC, and sR_tot_ with delta values of MLD may relate to the dynamic information provided by delta values. Previously, FWHM of the Hounsfield distribution was suggested to be associated with parenchymal or emphysematous heterogeneity [17]. We found significant correlations between FWHM and functional parameters of emphysema (e.g., RV%TLC) and obstruction (e.g., FEV_1_%VC) in expiratory but not inspiratory scans. From a pathophysiological standpoint, this may be explained by increasing differences between normal parenchyma and emphysematous areas in COPD patients compared to pulmonary healthy subjects. This effect may become more apparent during expiration due to increased geographic air trapping in patients with COPD.

TLC% and inspiratory volume showed only a weak correlation (0.38), despite the fact, that both parameters should measure the same volume. Beyond the weak correlation, the absolute values differed. One reason for this difference is the quantification process of the CT images. The software automatically ads a distance of 1 cm between the quantified lung volume and pleura to eliminate errors occurring through pleural irregularities. This led to a reduced total lung volume in the qCT when compared to lung function tests. Further, total lung volume was acquired in supine position in qCT rather than sitting TLC in body plethysmography. As shown previously, posture has an effect on measured lung volumes and thereby might have strengthened the named difference [33].

Our study has several limitations that must be considered. First, we did not perform spirometric triggering during CT. Thereby, we cannot verify that all patients strictly followed the breathing commands. There was a mean volume difference of 1 liter between maximal inspiration and expiration, and we believe that our data are representative of functional inspiration and expiration. Second, since we only included patients with clinical indications for unenhanced chest CT, severe stages of COPD might be overrepresented in our cohort. Third, the number of subjects included in our study was rather small.

The strength of our study is the systematic evaluation of expiratory values, stand-alone delta values, and their respective correlations with lung function testing. As mentioned before, previous studies have correlated qCT parameters and lung function tests. But they did not take the expiratory and stand-alone delta values into account as we did in this work. Moreover, we focused on lung function parameters acquired by body plethysmography, which has advantages compared to traditional spirometry: While RV and TLC cannot be measured by spirometry, both are altered in COPD due to the loss of elastic recoil, airway closure, and hyperinflation of the lung [34]. Our study mainly addressed the relation of qCT and spirometric parameters. As stated initially, spirometry might not be ideal for COPD characterization. Nevertheless, we believe that the coherence of image data and lung function parameters is worth pointing out and need to be shown as a foundation for further research. Therefore, future studies should evaluate a qCT-based COPD characterization also considering expiratory and delta values. This would be particularly interesting in context of small airway disease. However, spirometry and body plethysmography used in our investigation are not ideal techniques and therefore further investigation including, for example, impulse oscillometry is warranted.

Overall, our study confirms three major presumptions. First, quantified CT parameters correlate with lung function parameters beyond the commonly used FEV_1_ and VC. Second, a single acquisition in maximum inspiration alone is an incomplete approach for comparison of quantified CT parameters and functional lung parameters in COPD. Again, from a pathophysiological standpoint, these findings are related to the fact that COPD, as an *obstructive* lung disease, is most manifest at expiration. An additional scan in maximal expiration does not only provide a wider significant correlation profile but also allows the calculation of the delta value. These delta values have to be seen as an equivalent and discrete parameter. This becomes most evident for FWHM, with dynamic lung function parameters correlating significantly with delta and expiratory values, yet not with inspiratory values.

Consequently, the additional acquisition of an expiratory scan does not only provide a wider range of significant correlations with lung function parameters itself but also allows the calculation of the delta values. This does not only leads to more significant correlations to functional lung parameters but might also be important for future phenotyping of COPD with combined quantified CT and pulmonary function tests. Thereby, the expiratory and the delta values contain additional information and should be considered as mandatory correlation parameters in future studies.

## Figures and Tables

**Table 1:** Patient characteristics.

*n*	♂/♀	Age, years (range)	Size, cm (range)	Weight, kg (range)	Smokers/ex-smokers
46	26/20	66 ± 10 (44–87)	166 ± 7 (153–190)	76 ± 18 (34–117)	20/26

**Table 2:** Lung function parameters.

Parameter	Mean	SD	Range
VC (%)	70	23.3	22.2–128.6
FEV_1_ (%)	49.4	20.8	16.1–100.4
FEV_1_%VC	53.4	13	32.7–90.9
RV (%)	210.9	81.9	35.3–405.7
TLC (%)	125	31.6	54.4–205
RV%TLC	65	14.8	16.4–87.6
sR_tot_ (%)	337.4	241	47.8–1054.8

VC: vital capacity; FEV_1_: forced expiratory volume in one second; FEV_1_%VC: Tiffeneau index; RV: residual volume; TLC: total lung capacity; sR_tot_: specific total airway resistance.

**Table 3:** Quantified CT parameters.

Parameter	Mean		SD		Range	
	Inspiration	Expiration	Inspiration	Expiration	Inspiration	Expiration
Volume (ml)	5421	4417	1441	1279	2528 to 8541	1859 to 7792
MLD (HU)	−840	−803	48	66	−915 to −715	−910 to −663
FWHM (HU)	94	123	32	43	27 to 230	71 to 231
LAV (%)	12	9	15	14	0 to 50	0 to 50

MLD: mean lung density; FWHM: full width half max; LAV: low attenuation volume.

**Table 4:** Comparison between inspiratory and expiratory correlation coefficients.

Quantified CT parameter	Lung function parameter	Correlation inspiration	Correlation expiration	*z* value	*p* value
Volume	VC	0.0577	−0.2367	3.1409	0.0017
Volume	FEV_1_	−0.1314	−0.4643	3.631	0.0003
Volume	FEV_1_%VC	−0.3605	−0.5225	1.8699	0.0615
Volume	RV	0.3587	0.6466	−3.3049	0.001
Volume	TLC	0.3809	0.5503	−1.9779	0.0479
Volume	RV%TLC	0.1813	0.549	−4.0252	0.0001
Volume	sR_tot_	0.102	0.4345	−3.6152	0.0003
MLD	VC	−0.0194	0.1563	−1.9585	0.0502
MLD	FEV_1_	0.2186	0.4696	−2.9337	0.0033
MLD	FEV_1_%VC	0.4856	0.6376	−2.005	0.045
MLD	RV	−0.4421	−0.6362	2.4796	0.0132
MLD	TLC	−0.5098	−0.6378	1.7201	0.0854
MLD	RV%TLC	−0.2924	−0.6108	3.7492	0.0002
MLD	sR_tot_	−0.3061	−0.5225	2.5933	0.0095
FWHM	VC	0.0602	0.2715	−1.8312	0.0671
FWHM	FEV_1_	0.0918	0.4228	−2.9634	0.003
FWHM	FEV_1_%VC	0.1818	0.403	−1.9774	0.048
FWHM	RV	−0.1707	−0.416	2.1972	0.028
FWHM	TLC	−0.1718	−0.3402	1.4834	0.138
FWHM	RV%TLC	−0.0505	−0.4579	3.6813	0.0002
FWHM	sR_tot_	−0.0756	−0.3345	2.2735	0.023
LAV	VC	−0.2261	−0.346	3.1849	0.0014
LAV	FEV_1_	−0.3907	−0.4465	1.5801	0.1141
LAV	FEV_1_%VC	−0.4002	−0.336	−1.7761	0.0757
LAV	RV	0.5293	0.5748	−1.3982	0.1621
LAV	TLC	0.4354	0.405	0.8695	0.3846
LAV	RV%TLC	0.4527	0.5015	−1.4274	0.1535
LAV	sR_tot_	0.5235	0.5602	−1.1266	0.2599

MLD: mean lung density; FWHM: full width half max; LAV: low attenuation volume; VC: vital capacity; FEV_1_: forced expiratory volume in one second; FEV_1_%VC: Tiffeneau index; RV: residual volume; TLC: total lung capacity; sR_tot_: specific total airway resistance.

## Supplementary Materials

Supplementary Materials Figure E1: correlations of mean lung density (MLD) and forced expiratory volume in one second (FEV_1_) for inspiratory, expiratory, and calculated delta values. Figure E2: voxel-density histogram from a qCT. Table E1: correlation of quantified CT and lung function parameters for inspiration scan. Table E2: correlation of quantified CT and body plethysmography parameters for expiration scan. Table E3: correlation of quantified CT and body plethysmography parameters for delta values.
