# Supplementary material for: Time to Exhale: Additional Value of Expiratory Chest CT in Chronic Obstructive Pulmonary Disease
Source: Can Respir J. 2018 Mar 4;2018:9493504. doi: 10.1155/2018/9493504 (PMC5857310; doi:10.1155/2018/9493504)
Supplement: Supplementary Materials — Figure E1: correlations of mean lung density (MLD) and forced expiratory volume in one second (FEV1) for inspiratory, expiratory, and calculated delta values. Figure E2: voxel-density histogram from a qCT. Table E1: correlation of quantified CT and lung function parameters for inspiration scan. Table E2: correlation of quantified CT and body plethysmography parameters for expiration scan. Table E3: correlation of quantified CT and body plethysmography parameters for delta values. [file 9493504.f1.pdf]

Time to exhale: additional value of expiratory chest CT in chronic obstructive pulmonary disease

Joshua Gawlitza; Frederik Trinkmann, MD; Andreas Fischer, MD; John W. Nance, MD, Claudia Henzler, MD; Joachim Saur, MD; Ibrahim Akin, MD; Martin Borggrefe, MD; Stefan O. Schoenberg, MD; Thomas Henzler, MD for the CType investigators

Supplemental Digital Content

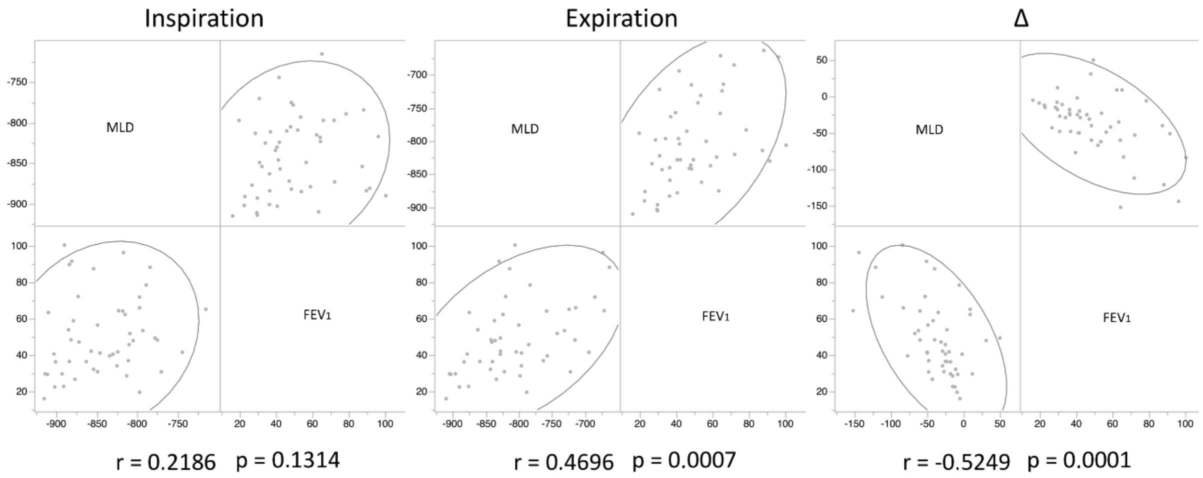

Figure E1: correlations of mean lung density (MLD) and forced expiratory volume in one second (FEV_1) for inspiratory, expiratory and calculated delta values.

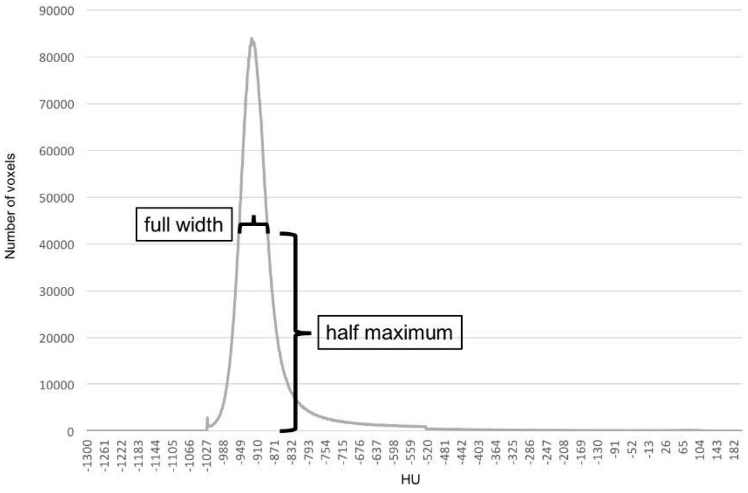

Figure E2: voxel-density histogram from a qCT. The number of voxels are represented on the y-axis while their HU values are on the x-axis. The full-width-half-max (FWHM) is the full width at the half maximum of this histogram.

Table E1: Correlation of quantified CT and lung function parameters for inspiration scan

Quantified CT parameter	lung function parameter	Correlation	Lower 95% CI	Upper 95% CI	p-value
Volume	VC	0.0577	-0.2272	0.3335	0.6938
Volume	FEV_1	-0.1314	-0.3979	0.1555	0.3681
Volume	FEV_1%VC	-0.3605	-0.5826	-0.0882	0.0109
Volume	RV	0.3587	0.083	0.5834	0.0123
Volume	TLC	0.3809	0.1117	0.5981	0.0069
Volume	RV%TLC	0.1813	-0.1052	0.4401	0.2125
Volume	sR_{tot}	0.102	-0.1845	0.3725	0.4858
MLD	VC	-0.0194	-0.2989	0.2633	0.8949
MLD	FEV_1	0.2186	-0.0667	0.4708	0.1314
MLD	FEV_1%VC	0.4856	0.2367	0.6747	0.0004
MLD	RV	-0.4421	-0.6452	-0.1806	0.0017
MLD	TLC	-0.5098	-0.6918	-0.2669	0.0002
MLD	RV%TLC	-0.2924	-0.53	-0.0122	0.0415
MLD	sR_{tot}	-0.3061	-0.5408	-0.0273	0.0324
FWHM	VC	0.0602	-0.2248	0.3357	0.6811
FWHM	FEV_1	0.0918	-0.1944	0.3636	0.5304
FWHM	FEV_1%VC	0.1818	-0.1048	0.4405	0.2113
FWHM	RV	-0.1707	-0.4338	0.1192	0.2461
FWHM	TLC	-0.1718	-0.4321	0.115	0.238
FWHM	RV%TLC	-0.0505	-0.3271	0.234	0.7303
FWHM	sR_{tot}	-0.0756	-0.3494	0.2101	0.6057
LAV	VC	-0.2261	-0.4769	0.0589	0.1183
LAV	FEV_1	-0.3907	-0.6054	-0.1231	0.0055
LAV	FEV_1%VC	-0.4002	-0.6125	-0.1341	0.0044
LAV	RV	0.5293	0.2885	0.7071	0.0001
LAV	TLC	0.4354	0.1757	0.6384	0.0018
LAV	RV%TLC	0.4527	0.1965	0.651	0.0011
LAV	sR_{to}	0.5235	0.2841	0.7014	0.0001

MLD: mean lung density, **FWHM**: full width half max, **LAV**: low attenuation volume, **VC**: vital capacity, **FEV_1**: forced expiratory volume in one second, **FEV_1%VC**: Tiffeneau index, **RV**: residual volume, **TLC**: total lung capacity, **sR_{tot}**: specific total airway resistance.

Table E2: Correlation of quantified CT and body plethysmography parameters for expiration scan

Quantified CT parameter	lung function parameter	Correlation	Lower 95% CI	Upper 95% CI	p-value
Volume	VC	-0.2367	-0.4856	0.0476	0.1015
Volume	FEV_1	-0.4643	-0.6594	-0.2106	0.0008
Volume	FEV_1%VC	-0.5225	-0.7008	-0.2829	0.0001
Volume	RV	0.6466	0.444	0.7863	<.0001
Volume	TLC	0.5503	0.3183	0.7201	<.0001
Volume	RV%TLC	0.549	0.3166	0.7191	<.0001
Volume	sR_{tot}	0.4345	0.1747	0.6378	0.0018
MLD	VC	0.1563	-0.1307	0.419	0.2837
MLD	FEV_1	0.4696	0.217	0.6632	0.0007
MLD	FEV_1%VC	0.6376	0.4342	0.7791	<.0001
MLD	RV	-0.6362	-0.7794	-0.4297	<.0001
MLD	TLC	-0.6378	-0.7792	-0.4345	<.0001
MLD	RV%TLC	-0.6108	-0.7612	-0.3979	<.0001
MLD	sR_{to}	-0.5225	-0.7007	-0.2829	0.0001
FWHM	VC	0.2715	-0.0105	0.5135	0.0592
FWHM	FEV_1	0.4228	0.1607	0.6292	0.0025
FWHM	FEV_1%VC	0.403	0.1374	0.6146	0.0041
FWHM	RV	-0.416	-0.6261	-0.1495	0.0033
FWHM	TLC	-0.3402	-0.5672	-0.0653	0.0168
FWHM	RV%TLC	-0.4579	-0.6548	-0.2029	0.0009
FWHM	sR_{tot}	-0.3345	-0.5628	-0.0589	0.0188
LAV	VC	-0.346	-0.5716	-0.0718	0.0149
LAV	FEV_1	-0.4465	-0.6465	-0.189	0.0013
LAV	FEV_1%VC	-0.336	-0.5639	-0.0605	0.0183
LAV	RV	0.5748	0.3474	0.7383	<.0001
LAV	TLC	0.405	0.1397	0.616	0.0039
LAV	RV%TLC	0.5015	0.2564	0.6859	0.0002
LAV	sR_{tot}	0.5602	0.3312	0.7269	<.0001

MLD: mean lung density, **FWHM**: full width half max, **LAV**: low attenuation volume, **VC**: vital capacity, **FEV_1**: forced expiratory volume in one second, **FEV_1%VC**: Tiffeneau index, **RV**: residual volume, **TLC**: total lung capacity, **sR_{tot}**: specific total airway resistance.

Table E3: Correlation of quantified CT and body plethysmography parameters for delta values

Quantified CT parameter	lung function parameter	Correlation	Lower 95% CI	Upper 95% CI	p-value
Volume	VC	0.4261	0.1646	0.6316	0.0023
Volume	FEV_1	0.4466	0.1892	0.6466	0.0013
Volume	FEV_1%VC	0.1645	-0.1223	0.426	0.2585
Volume	RV	-0.3411	-0.57	-0.0631	0.0177
Volume	TLC	-0.1712	-0.4316	0.1156	0.2396
Volume	RV%TLC	-0.4868	-0.6755	-0.2382	0.0004
Volume	sR_{tot}	-0.4515	-0.6501	-0.195	0.0011
MLD	VC	-0.2875	-0.5262	-0.0068	0.0452
MLD	FEV_1	-0.5249	-0.7024	-0.2859	0.0001
MLD	FEV_1%VC	-0.4814	-0.6717	-0.2315	0.0005
MLD	RV	0.5291	0.2883	0.7069	0.0001
MLD	TLC	0.452	0.1957	0.6505	0.0011
MLD	RV%TLC	0.6728	0.483	0.8022	<.0001
MLD	sR_{tot}	0.507	0.2634	0.6899	0.0002
FWHM	VC	-0.3059	-0.5406	-0.0271	0.0325
FWHM	FEV_1	-0.4784	-0.6695	-0.2278	0.0005
FWHM	FEV_1%VC	-0.3613	-0.5832	-0.0891	0.0108
FWHM	RV	0.3873	0.1159	0.6049	0.0065
FWHM	TLC	0.2866	0.0058	0.5254	0.0459
FWHM	RV%TLC	0.5673	0.3404	0.7318	<.0001
FWHM	sR_{tot}	0.3756	0.1055	0.594	0.0078
LAV	VC	0.3788	0.1093	0.5965	0.0073
LAV	FEV_1	0.1079	-0.1787	0.3776	0.4606
LAV	FEV_1%VC	-0.327	-0.557	-0.0505	0.0218
LAV	RV	-0.0366	-0.3174	0.2501	0.8049
LAV	TLC	0.2137	-0.0718	0.4669	0.1404
LAV	RV%TLC	-0.068	-0.3427	0.2173	0.6423
LAV	sR_{tot}	-0.0077	-0.2883	0.2741	0.958

MLD: mean lung density, **FWHM**: full width half max, **LAV**: low attenuation volume, **VC**: vital capacity, **FEV_1**: forced expiratory volume in one second, **FEV_1%VC**: Tiffeneau index, **RV**: residual volume, **TLC**: total lung capacity, **sR_{tot}**: specific total airway resistance.
